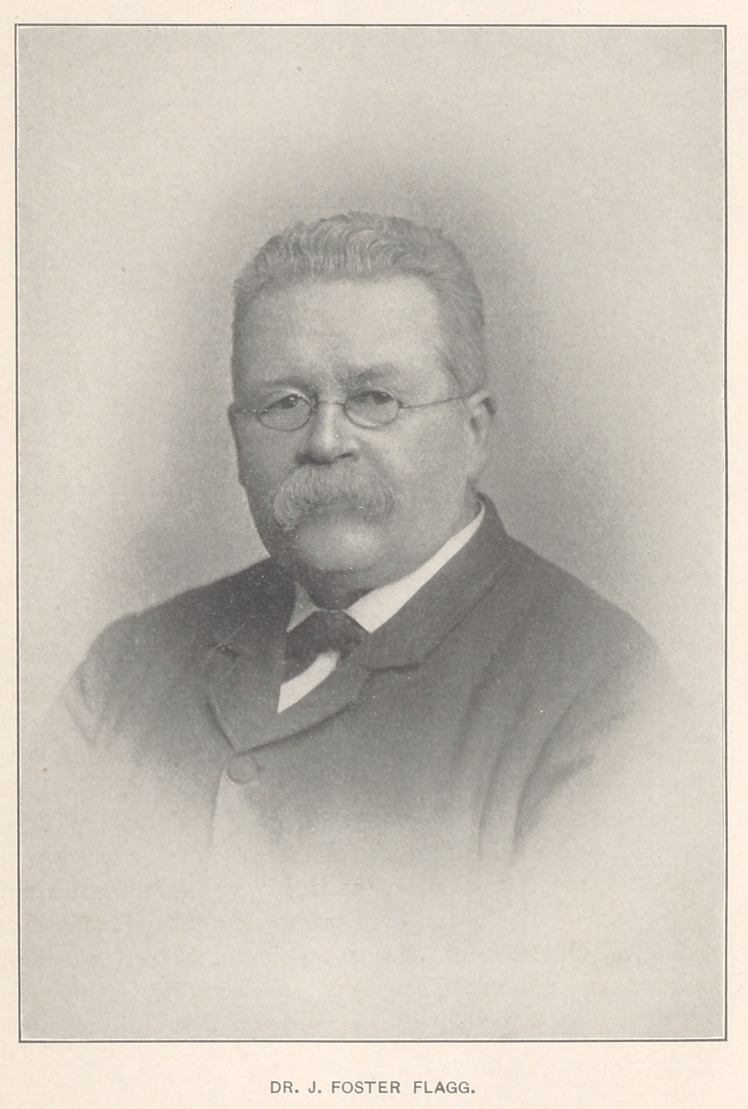# J. Foster Flagg, D.D.S.

**Published:** 1904-01

**Authors:** William H. Trueman

**Affiliations:** Philadelphia


DR. J. FOSTER FLAGG.

### Biographical Sketch.

### J. FOSTER FLAGG, D.D.S.

BY WILLIAM H. TRUEMAN, D.D.S., PHILADELPHIA.

Died, at his residence, Swarthmore, Pa., on the evening of November 25, 1903, Dr. J. Foster Flagg, after a long and painful illness borne with manly fortitude.

Dr. Josiah Foster Flagg, the only son of Dr. John Foster Brewster Flagg and Miss Mary Waterman Jackson, was born at Providence, R. I., October 15, 1828. He was a grandson of Josiah Flagg, the first native-born American dentist, and the last male descendent of that branch of the Flagg family. The family history dates back to Thomas Flagg, supposed to be a native of Ireland, who arrived at Watertown, Mass., in 1642. It was through his great-grandfather, Lieutenant-Colonel Josiah Flagg, that the family became so closely identified with the rise and progress of dental science in America. Josiah Flagg, then a lad of about eighteen years, was a private in the Elliott regiment, of which his father was an officer, when the American and French troops were encamped in winter quarters near Providence, R. I., 1781-82. The war was practically over, and Joseph Le Maire, a surgeon-dentist from Paris, serving as a surgeon with the French contingent, resumed the practice of his profession. He and Lieutenant-Colonel Flagg became intimate, and the opportunity thus offered for his son to learn this new profession from so skilful a master was promptly embraced. Young Flagg proved an apt student, and achieved a fair measure of success. His eldest son, Josiah Foster Flagg, was educated for the medical profession, but on the death of his father, in 1816, entered upon dental practice.

In 1797 Josiah Flagg married, as his second wife, Miss Eliza Brewster, a direct descendant of the sixth generation from Elder Brewster, who came over in the "Mayflower" as leader of the first contingent of Pilgrim Fathers, thus uniting these two old colonial families. Dr. Flagg's father, J. F. B. Flagg, was the only son of this marriage. His father dying in 1816, when he was only twelve years of age, his education was directed by his elder brother, who exercised over him a fatherly care, and in due time taught him the art and science of dental surgery. On reaching manhood he moved to Providence, R. I., and shortly after married Miss Mary Waterman Jackson, daughter of a prominent citizen of that town. Dr. J. Foster Flagg was their only son. The early education of the latter was received at the schools of that place, and later at a school in Boston conducted by Bronson Alcott. In 1842 his father removed to Philadelphia, and soon became intimate with the more prominent Philadelphia dentists, and was an important factor in inaugurating the movement which resulted in making Philadelphia an important dental educational centre.

On leaving school, Dr. Flagg entered the Jefferson Medical College of Philadelphia, but on account of being under age did not graduate. About 1849 the California gold excitement electrified the country. He had not as yet "settled down," and, with a desire to see the world, he immediately set out for the far distant gold-fields, taking with him an assortment of dental and medical instruments and drugs. The excitement appealed to his active ardent nature, and in due time he arrived at the expected "Eldorado." While there he had varied and exciting experiences. At one time at the gold-diggings, again as a cow-boy on the plains, and later engaged in the first attempt to impound the waters of the mountain-streams for use in mining and irrigation, he and his colaborers laid the foundation of that which has done so much to develop the industries of that region. It might well be called "a wild life," but J. Foster Flagg knew how to take care of himself; his home training was not forgotten, and his surroundings developed in him a manliness, mentally and physically, that served him well in later years. In camp he was a leader; there was that about him that commanded respect while inviting comradeship; and now and again his well-trained muscles were called upon to administer convincing arguments to those of the unruly element, that he was not to be trifled with.

The dental and medical knowledge acquired before leaving home now came into use, and he soon became a much-sought physician, surgeon, and dentist in treating the disease and accidents of camp life. The much dreaded Asiatic cholera played sad havoc in many camps. On its approach Dr. Flagg instituted strict sanitary precautions, and as a prophylactic adopted the free use of acids, with the result that the disease did not prevail where these precautions were observed. For treatment he advocated giving the sufferers all the water they cared to drink, well dosed with acid, opium only when needed. Pickles were among the camp luxuries, and the surplus vinegar was at times the only obtainable acid. It proved very efficient, and was carefully hoarded, being considered worth "its weight in gold."

The methods of gold-mining then practised required an abundance of water. The supply from the mountain-streams was very irregular, at times deficient, and at other times destructive in its abundance. Dr. Flagg, with a few others, conceived the idea of impounding these streams so as to secure a more reliable supply. While at work solving the engineering problems involved in building a dam to store up water for mining and agricultural purposes, word reached him that his mother was seriously ill, and had a yearning to see once more her only son. His maternal love overcame his ambition, and he promptly decided to at once start for home. This proved a very great financial loss. While he and his partners were well satisfied that their work would prove successful, and in the end profitable, and had embarked their all in the enterprise, it was generally distrusted, and considered a wild scheme. While the desirability of impounding and holding for a time of need the surplus water of the rainy season was fully recognized, its practicability was doubted, and it was prophesied that the first freshet would wipe out all their labor. This made it difficult for him to sell out his interest to advantage; but as he had been there nearly seven years, and was feeling homesick, he decided to leave the Pacific coast for good, and therefore desired to close out all business interests. To do so he was compelled to accept for his share in the enterprise less than he had put into it, and very much less than it was worth, as the sequel proved. His engineering plans proved successful. He had well studied the forces the dam would encounter, and so well planned to resist them that it withstood many freshets, and as a venture was profitable beyond expectation. Enlarged and improved, it continues useful to this day.

His next care was the home voyage. He was informed that a vessel was all ready to sail from San Francisco, but waited to obtain a competent medical officer, and it was suggested to him that his medical experience and reputation in camp might obtain him the position. He applied, and his application was endorsed by some of his friends. The examination was brief. "Can you treat cholera?" "I can," was the prompt and emphatic answer. It was enough. He was engaged for the voyage and given his passage for his services, with orders to as quickly as possible provide an ample medical chest at the company's expense. So frequently and so fatally had the dread disease appeared on former voyages, that no vessel would sail without a physician; the position was not sought after, and this vessel had waited days to supply this need when Dr. Flagg applied. In relating the incident he confessed that the situation was embarrassing. He had no fear of cholera, unless he himself should be the victim, and he inwardly prayed that there should be no other diseases, and but little of that. It was his only chance to get home quickly. The passenger-list was full, and no other vessel was expected to sail for a month. Furthermore, the free passage well suited his finances. He promptly directed and enforced such proper sanitary precautions as were possible in an overcrowded passenger-ship, and was the one man in all that ship's company whose orders were willingly accepted and promptly executed. These proved efficient. That vessel was one of very few sailing from San Francisco to Panama at this time which, on arriving at its destination, was able to report "no serious sickness and no deaths."

Once more at home the question of his life's vocation was seriously considered. He finally decided to adopt his father's profession, and entering the Philadelphia College of Dental Surgery, graduated from that institution at its fourth annual commencement, February 29, 1856, in the same class with his distinguished colleague, the late Professor James E. Garretson.

For a few years he practised in New Jersey, but returned to Philadelphia in 1860, and located with his father at 1112 Arch Street. Fortuitous circumstances assisted him to quickly acquire a satisfactory practice. His genial manners, his professional skill, and his gentleness in operating gave him a firm hold upon his patients. Among these was a large number of school children, whose gratitude he earned by refusing to make appointments with them on Saturday. He contended it was their holiday, and should not be broken into even for an hour; the care of their teeth was fully as important as their education, and should be done in other time than theirs.

October 31, 1861, he married Miss Mary Craft, who survives him.

By prudent living and good business management during his active life, Dr. Flagg was able to retire when advancing years made professional duties a burden. At his comfortable, pleasantly situated country home at Swarthmore, with congenial neighbors, his children and grandchildren close at hand, relieved from all care, he enjoyed for a few years a well-earned rest. He was not, however, idle. He continued to manufacture the plastic filling-materials he had done so much to improve, and continued his experiments looking to a still further elimination of their defects, until the inroads of disease compelled him to stop.

He leaves two daughters,—Mary, the wife of Dr. James Price, of Swarthmore, and Lillie, wife of Professor Gummere, of Ursinus College, Collegeville, Pa.

Dr. J. Foster Flagg entered the dental profession determined to succeed; he was energetic, enthusiastic, and a tireless worker. He possessed in full measure the true professional spirit, and held, taught, and practised that every man's interests were best served when each tried to help the other. He promptly became a contributor to periodical dental literature, addressing himself more particularly to those problems usually termed "practical," those which immediately concern a dentist's daily work. His first contribution was upon the construction of artificial teeth (*Dental News Letter,* vol. x., October, 1856, page 209), dealing with the artistic arrangement, and referring to points he had observed to be frequently overlooked. He was a keen observer, quick to appreciate the relation of cause and effect, and resourceful in overcoming the many difficulties constantly taxing the abilities of a dental practitioner. He *knew* what he *did* know, and expressed his convictions with positiveness and confidence, making, however, no pretensions regarding matters of which he was not sure. "I don't know," was with him a frequent expression when conversing upon professional matters; "I think," "It may be so," "I don't understand it," "That is out of my line," etc., were his usual comments upon unsolved problems; but, regarding those which he felt had been solved, his emphatic "I know" admitted of no question and tolerated no doubt. There was in this no egotism, it indicated absolute confidence, nothing more. While holding in profound respect the conclusions of others, especially those which had become crystallized into accepted theories and generally considered safe guides in dental practice, he did not permit them to override or obscure his own observations. He was ever open to new ideas, to consider new theories and new methods upon their intrinsic merits rather than the reputation of their authors. His judgment on these was usually quickly rendered, and generally accurate.

Dr. Flagg began his career as a teacher by accepting the chair of Institutes of Dentistry, in the first faculty of the Philadelphia Dental College at its organization in the spring of 1863, and he outlived them all. The title of this chair was changed at the beginning of the sixth session, 1868-69, to that of Dental Pathology and Therapeutics, a change of name only. He resigned at the close of the seventh session in order to devote his time more fully to private practice, continuing, however, his connection with the college as clinical instructor. With the opening of the seventeenth session, 1879-80, he resumed his old position and continued to lecture until the close of the session of 1895-96, when he finally retired. During this period the Philadelphia Dental College made a decided advance. About 1887 it united with the Medico-Chirurgical College in the erection of a new building for joint occupancy, in order to secure more room and more convenient arrangement to accomodate their constantly increasing classes. Dr. Flagg had much to do with designing the new structure, and skilfully planned the various class- and clinic-rooms to secure the largest capacity without sacrifice of comfort and convenience. During the erection he superintended the details of construction, meeting and solving the many problems that arose as the work progressed. For this he was eminently qualified. He knew what was needed, and his mechanical ingenuity suggested novel expedients which added much to the usefulness of the finished structure, which proved well adapted to its intended use.

As a teacher he displayed marked ability. He had a personal magnetism that attracted and retained the students' attention; he was earnest, enthusiastic, spoke with energy and emphasis, and interspersed in his remarks witty sayings and anecdotes, so appropriate and well told that they served to firmly fix the facts presented in the minds of his hearers. His lectures were not desultory reading of text-books; on the contrary, they were original, well connected, and interesting discourses, and recitals of personal experiences having a direct bearing upon the vocation with which the students were most concerned.

Wherever possible he enforced the spoken word by demonstrating before his class, and constructed for this purpose many ingenious models, among them a rudely formed skeleton of the head, with the teeth and jaws in position. This proved an admirable arrangement for illustrating the position to be assumed in operating, arranging napkin, bandages, etc., and the most convenient way of performing various dental operations. This was duplicated and patented in England as something new more than a score of years after Dr. Flagg had introduced it to his classes.

He had the happy faculty of making himself one with his students without sacrificing the dignity of a teacher. He was approachable, invited their friendship, and made their interests his.

As a writer Dr. Flagg contributed to dental periodical literature all through his professional life. He wrote as he spoke, with emphasis and vigor. His style was quite original, and while it at times lacked scholarly dignity, it conveyed unerringly the writer's thought. He was inclined to be epigrammatic, while wit and sarcasm, pointed, yet so refined as to be thoroughly enjoyed even by those who felt its shafts, flowed freely from his pen. His most notable production is his work on "Plastics," a work that merits a place in all dental libraries.

Dr. Flagg can hardly be considered a professional society man. He could not forget that his father and uncle were compelled to forego membership in the American Association of Dental Surgeons for no other fault than that of declining to sign away their right of using their own judgment in matters of practice. He noted with keen regret that the professional societies held, with all the tenacity of religious bigots, to the accepted tenets of the day. As he once remarked to the writer, he seemed to have been born a professional heretic, and from first to last of his professional career was the subject of adverse criticism, and at times of reproach. He bore it all, however, in good part, and lived to see many of his derided ideas accepted and adopted, and would now and again remark, as he noted in society discussion the once denounced suggestions advanced as good practice, "They are getting up to me; they will be there after a while." Notwithstanding, however, he keenly regretted the spirit of intolerance so frequently displayed in dental societies, and regarded it, as all thoughtful men must do, as a hinderance to real progress. While he frequently attended dental society meetings, thoroughly enjoyed them, and took an active part whenever present, and felt and knew that his remarks were enjoyed and appreciated, he felt more free as a visitor than as a member. That a man remains placid, and replies playfully to adverse criticism, does not imply that he enjoys it. Dr. Flagg did not. He held his membership in dental societies ever ready to slip his moorings to escape expulsion for unwittingly transgressing some part of the *creed* the society itself might shortly expunge.

Early in his career as a dentist Dr. Flagg was impressed with the importance of saving all of the natural teeth that could by any possible means be kept useful and comfortable. He appreciated the difficulties attending the manipulation of gold in teeth badly broken down, and observed that the much decried amalgam seemed to be especially useful in such cases. The expression so frequently used at that time, "Any tooth worth saving should be filled with gold," did not appear to him as a good motto for a dentist who wished to do the best for his patient. To him it seemed more reasonable that any tooth that could be made comfortable and was useful was worth saving, and he realized that this included many teeth that could not be filled with gold by even the most expert operators. The so-called "amalgam war" had just closed; notwithstanding that, however, the leading lights of the dental profession were not converted; they still believed that amalgam was a vile stuff to place in carious human teeth, and had not yet learned that many of the ills credited to it were due to improper or imperfect treatment of the tooth preparatory to inserting the filling. Dr. Flagg's father and uncle, while bitterly denouncing amalgam, quite as bitterly denounced the American Association for provoking the controversy, holding that it had no right to impose restrictions upon its members in their efforts to benefit their patients. Dr. Flagg had been taught to avoid amalgam, many of his professional associates were opposed to it, and he naturally had a prejudice against it. He had not been long in practice, however, when he became convinced that in many cases it was the only available means of prolonging the usefulness of teeth important to the comfort of their owners. He resolved that inasmuch as he alone was responsible for the results of his operations, and that this responsibility could not be shared by those who assumed to dictate what was and what was not in accordance with professional probity, he would be guided in all such matters by his own experience and judgment.

Laying aside as far as possible preconceived notions, he addressed himself to the problem of tooth-saving. He was thus led to look upon amalgam as a good thing to use when nothing better was available. He carefully noted its many defects, and by a long series of careful observations and experiments sought their elimination. Conducted as this research was, progress was necessarily slow; it was, however, sure. He first noted the differing behavior of amalgams made of various alloys, and endeavored to ascertain the part played by their several components. From his professional associates he selected a number whom he knew to be skilful, unprejudiced, careful observers, men in whose judgment he had confidence, and whose fields of labor were widely separated, to test the various alloys he experimentally compounded, asking from them reports of their behavior as fillings after an interval of several years. They were requested to carefully prepare a record of the position, surroundings, and the circumstances attending the insertion of each filling; to place them as far as possible where they could be frequently seen and their condition noted. Especial attention was asked regarding tooth-saving, integrity, and color. Dr. Flagg kept a record of the formula of these alloys, and full particulars of the treatment they received before they left his hands. When reports from these came back to him after an interval of some years, he was able to collate the experience of many observers, working under varied conditions, and to know far more than could possibly have been learned by laboratory experiments alone. In many cases reports were, after a longer interval, revised. An alloy that was pronounced satisfactory after two years' observation might be condemned a few years later. After years of similar experimentation he began to learn the varied properties of the available metals, how these properties were modified by alloying, and to select and properly proportion them to produce a desired result. When this point was reached, about 1881, he published the first edition of his work entitled, "Plastics and Plastic Fillings." In this work he embodied the results of his labors to date, and by subsequent editions and corrections he has kept the profession fully informed of progress made. None but those close to Dr. Flagg can appreciate the vast labor these researches, continued more than twoscore years, involved, the care with which they were conducted, or how cautiously the results were from time to time announced. Now and again he announced to his friends, "I have done with it; I have not reached the end, but I have done all

I can; let some one else finish it." He continued, however, his efforts to improve this class of fillings until the very end of his long and useful life. In his last interview with the writer, but a few months before his death, he said that he had just completed some experiments by which he thought he had obtained an amalgam that preserved its color better, and a gutta-percha better able to withstand wear; as soon as he was satisfied with the tests then in progress he intended the profession to have the benefit of it. That was his last effort. He died in harness.

As an unlooked for outcome of these researches the profession was startled about a quarter of a century ago by a boldly announced "New Departure." While making these researches upon amalgam, Dr. Flagg noted that amalgam seemed to have tooth preservative properties apart from those due to its plasticity. Upon examining further in this direction, he observed that in mouths where gold fillings were apt to prove temporary only, decay quickly recurring around the filling or at its cervical margin, amalgam often proved more lasting and more effective in arresting decay. He further noted that with a class of teeth universally termed "soft," teeth that seemed prone to decay, and in which gold fillings required frequent renewal, teeth that dentists in general consider as doomed to be early lost, did far better when filled with amalgam. He still further observed that in cases of recurring decay, if the new decay was removed and the defect repaired with amalgam, the operation was usually more permanent than when the defective filling was removed and replaced with gold, or when the repair was made with gold. These results were unlooked for, and were not inexplicable by any theory then in vogue with the dental profession. It had been before observed that tin was in some cases a better decay arrester than gold, and that gutta-percha was equally effective, if not more so. At first this was explained by the softness of tin and gutta-percha permitting a better adaptation to the cavity walls, but later and more searching observations established that these two tooth-filling materials seemed to be effective in cases where it was known that the cavity adaptation was imperfect, while gold often failed in spite of the best adaptation an expert operator was able to make, and in fillings that seemed perfect.

About this time the late Dr. Stewart B. Palmer, of Syracuse, N. Y., and the late Dr. Henry S. Chase, of St. Louis, Mo. (*Dental Cosmos*, vol. xviii., 1876, pages 244 and 352), both of whom were in correspondence and were working with Dr. Flagg in these investigations, suggested the electro-chemical theory as an explanation. They, indeed, contended that under certain conditions at times present in the oral cavity a filling in a tooth becomes a galvanic battery whose energy depends upon the relation, electrically, between the substance of the tooth and the material of the filling. The farther they are apart, potentially, the more energetic this battery becomes. The result of this electric energy, they contended, was a changed condition of that portion of the oral fluids immediately at the junction of the tooth and the filling, by which they became acid and tooth-destroying. Inasmuch as gold and the substance of the tooth (for convenience in this discussion termed "dentos") are widely separated, on the electro-potential scale, while dentos, tin, amalgam, the cements, and gutta-percha are in the order named closely related, this was presented as an explanation of the mystery. Dr. Flagg made no pretention to knowledge of these intricate matters. The explanation was plausible. He therefore called to his aid scientists well qualified to investigate the matter, and was by them informed that the theory was in accord with recognized principles of electro-chemistry.

These three investigators were now convinced that the failure of gold fillings was not due to defective manipulation, nor yet to an inherent weakness of the teeth themselves, as had heretofore been so strenuously held. Manipulative ability had failed to make gold the tooth-saver the dentist needed in so many cases, and, they felt, it was high time to take a new departure by abandoning this cure-all and using in its place something else for those teeth in which it had so signally failed. The real question was not gold or plastics. They admitted that there was nothing better than gold for all cases where it effectively arrested decay, but contended that its continued use in places where general experience taught it was not effective was not good practice. As was well known, so little are some teeth prone to decay that any kind of a filling will effectively arrest its destruction, while, on the other hand, the best efforts of an expert fails to do more than retard it in others. The first class needs but little help, the second, all the help dental science can give them. Hence the first article of the so-called "New Departure Creed," "In proportion as teeth need saving, gold is the worst material to use," the worst material because by its presence, to a greater degree than does any other tooth-filling material, it brings about a local condition favorable to tooth destruction. That was the theory of the new departure.

Dr. Flagg made a forceful presentation of the subject in an address delivered at a special meeting of the New York Odontological Society, November 20 and 21, 1877. He was well qualified for the task. He felt he was right, and spoke with earnestness and energy. He well knew that the views expressed would encounter a strong opposition, and invite to himself and his colleagues adverse criticism. His purpose was a laudable one. It was to get the profession out of a rut, a slavish following of the old maxim that gold was the only filling-material a respectable dentist should use, and to elevate from the realms of quackery the much abused yet useful plastics. Notwithstanding the tempest his address aroused, it accomplished its purpose, and stimulated a series of improvements in all these plastic fillings that has given them a wider field and increased usefulness. For this the profession owes Dr. Flagg a debt of gratitude.

The value of Dr. Flagg's services in introducing improved formulas for dental alloys and new methods of making and preparing them for use,—and in advocating the use of the non-metallic plastics and acting as their champion on their advent into respectable dental practice in this country, will be more and more appreciated as time goes on. The profession may disregard the theories of the "New Departure," but there is, nevertheless, an unmistakable growing tendency towards the practice he advocated, and a general recognition that it tends to greater success in tooth-saving. Dr. Flagg had reason for now and again facetiously remarking, "They are getting there; they will be up to me after a while."

What an honored place does the three generations of this family occupy in the annals of dentistry in America! The grandfather, the pioneer native-born American dentist; his two sons, distinguished alike as practitioners, teachers, and investigators, who have made the way easier for those who follow; while the grandson's earnest efforts to increase the usefulness of the profession promises to revolutionize the practice of the science. With the death of Josiah Foster Flagg the chapter ends.

## Figures and Tables

**Figure f1:**